# Proteomics Revealed That Mitochondrial Function Contributed to the Protective Effect of Herba Siegesbeckiae Against Cardiac Ischemia/Reperfusion Injury

**DOI:** 10.3389/fcvm.2022.895797

**Published:** 2022-07-06

**Authors:** Xiaohong Wei, Yuzhuo Wu, Haie Pan, Qian Zhang, Ke He, Guiyang Xia, Huan Xia, Sheng Lin, Hong-Cai Shang

**Affiliations:** ^1^Key Laboratory of Chinese Internal Medicine of Ministry of Education and Beijing, Dongzhimen Hospital, Beijing University of Chinese Medicine, Beijing, China; ^2^Guangdong Provincial Key Laboratory of Chinese Medicine for Prevention and Treatment of Refractory Chronic Disease, Guangzhou, China

**Keywords:** Herba Siegesbeckiae, myocardial ischemia/reperfusion injury, mitochondrial electron transport chain, MnSOD acetylation, NLRP3 inflammasome

## Abstract

**Background:**

Myocardial ischemia/reperfusion (I/R) injury is the main obstacle to percutaneous coronary intervention, lacking effective therapeutic measures in a clinical setting. Herba Siegesbeckiae (HS) is a traditional herb with multiple pharmacological activities and evidence of cardiovascular protection. However, few data are available regarding the role of HS in cardiac I/R. This study aimed to explore the effect and underlying mechanism of HS aqueous extract on cardiac I/R injury.

**Materials and Methods:**

Herba Siegesbeckiae aqueous extract was prepared and analyzed by UHPLC-MS/MS. After intragastric administration of HS once daily for 7 days, male Sprague-Dawley rats were subjected to 30 min occlusion of the left anterior descending coronary artery followed by 120 min reperfusion to elicit I/R. Various parameters like myocardial infarction and apoptosis, 12-lead ECG and hemodynamics, cardiac morphology and myocardial enzymes, quantitative proteomics, mitochondrial ultrastructure and electron transport chain (ETC) function, oxidative stress and antioxidation, and NLRP3 inflammasome and inflammation were evaluated.

**Results:**

The chemical constituents of HS aqueous extract were mainly divided into flavonoids, diterpenoids, and organic acids. *In vivo*, HS aqueous extract notably alleviated myocardial I/R injury, as evidenced by a reduction in infarct size, apoptotic cells, and cardiac lesion enzymes; decline of ST-segment elevation; improvement of cardiac function; and preservation of morphology. Quantitative proteomics demonstrated that HS reversed the alteration in the expression of Adgb, Cbr1, Decr1, Eif5, Uchl5, Lmo7, Bdh1, Ckmt2, COX7A, and RT1-CE1 after I/R. In addition, HS preserved myocardial ultrastructure and restored the function of mitochondrial ETC complexes following exposure to I/R; HS significantly suppressed I/R-elicited increase of ROS, RNS, MDA, and 8-OHdG, restrained the acetylation of MnSOD, and recovered the activity of MnSOD; and HS reversed I/R-induced elevation of NLRP3 inflammasome and inhibited the release of inflammatory factors and pyroptosis.

**Conclusion:**

Herba Siegesbeckiae aqueous extract ameliorated cardiac I/R injury, which is associated with mitigating oxidative stress, suppressing NLRP3 inflammasome, and restoring mitochondrial function by regulating the expression of Adgb, Cbr1, Decr1, Eif5, Uchl5, Lmo7, Bdh1, Ckmt2, COX7A, and RT1-CE1.

## Introduction

Percutaneous coronary intervention is the main effective strategy to treat acute coronary syndrome (ACS) by recovering myocardial perfusion, which can save patients’ lives to a certain extent (1). However, the mortality due to ACS has not been reduced, mainly due to myocardial ischemia/reperfusion (I/R) injury and subsequent myocardial fibrosis and heart failure (2). Though the significance of conquering I/R has been recognized for decades, there are currently only a few effective therapeutic interventions applied in a clinical setting to prevent I/R (3).

The pathogenesis of myocardial I/R injury includes oxidative stress, inflammatory response, and mitochondrial dysfunction (4). Numerous studies have confirmed a link between mitochondrial malfunction, ROS, and chronic inflammation, whereas mitochondrial damage is thought to be essential for both ROS generation and inflammatory reactions (5, 6). First, mitochondria are the main source of cellular ROS (7, 8). When exposed to I/R, the mitochondrial electron transport chain is damaged and cannot transfer electrons from Complex I or Complex II through Complex III to Complex IV (cytochrome C oxidase), resulting in the generation of robust ROS from mitochondria (9, 10). Simultaneously, ROS cannot be scavenged in time and mitophagy is suppressed, leading to an increase in the number of damaged, ROS-generating mitochondria (5, 11).

Second, damaged mitochondria are necessary for the activation of the NLRP3 inflammasome, which has been proved in various cardiovascular diseases, including I/R injury (5, 12). NLRP3 inflammasome is a complex assembled by a sensor (NLRP3), an adaptor (apoptosis-associated speck-like protein with a caspase-recruitment domain, ASC), and an effector (caspase-1) in response to stress (13, 14). NLRP3 mainly localizes in the endoplasmic reticulum in the resting state, while NLRP3 and its adaptor ASC are redistributed to the perinuclear space during inflammasome activation, where they co-localize with mitochondria-associated endoplasmic reticulum and are involved in recruiting and activating pro-caspase-1 into caspase-1 (5). The activated caspase-1 then promotes the maturation and release of interleukin (IL)-1β and IL-18, and induces the synthesis of more amount of inflammatory factors, such as IL-6 and tumor necrosis factor (TNF)-α, thus leading to an inflammatory cascade response (15, 16). Moreover, activated caspase-1 can shear and activate Gasdermin D (GSDMD) protein, causing pyroptosis and aggravating mitochondrial damage (17). Recent studies in heart failure with preserved ejection fraction mice model and Sirt3-KO mice demonstrated that mitochondrial overacetylation promotes ASC aggregation into perinuclear mitochondria and NLRP3 inflammasome formation, implying that NLRP3 inflammasome activation is sensitive to mitochondrial function (18).

Herba Siegesbeckiae (HS) is a traditional Chinese medicine widely used for the treatment of rheumatic arthralgia associated with aching and weakness of loins and knees, as well as numbness of limbs (19). HS is considered to have a variety of pharmacological activities due to its multiple constituents. Previous studies mainly focused on HS ethanol extract, which could treat pressure overload-induced myocardial remodeling and global cerebral I/R damage (20). Additionally, HS aqueous extract was reported to exhibit antioxidant activity by inhibiting NOX2/ROS/NF-κB pathway and elevating SOD activity in rat knee osteoarthritis (20). However, little data are available regarding the chemical composition of HS aqueous extract and the effect of HS aqueous extract on myocardial I/R injury. The present study was launched to analyze the main components of HS aqueous extract, and to explore the effect and the underlying mechanism of HS on myocardial I/R injury.

## Materials and Methods

### Animals

Male Sprague–Dawley (SD) rats, weighing 220 ± 10 g, were purchased from Beijing HuaFuKang Biotechnology Co., Ltd with the certificate number SCXK (Beijing) 2019-0008. All animals received humane care in accordance with the National Institutes of Health Guide for the Care and Use of Laboratory Animals (NIH Publications No. 8023, revised 1978). Experimental procedures were approved by the Animal Care Committee of Dongzhimen Hospital Affiliated to the Beijing University of Chinese Medicine (No. 21-35).

### Qualitative UHPLC-MS/MS Analysis of Herba Siegesbeckiae Aqueous Extract

To prepare HS aqueous extract, 1 g of dry HS was soaked in 8 mL of water for 30 min, boiled for 30 min, and filtered. Then, 6 mL of water was added to the filtrate residue, boiled for 30 min, and filtered again. The filtrates were mixed and concentrated to 10 mg/mL by using a nitrogen evaporator. Then, 1 mL of 10 mg/mL HS aqueous extract was placed in a 1.5 mL centrifuge tube and centrifuged at 14,000 rpm for 5 min to prepare the stock solution for UHPLC-MS/MS analysis.

A Q Exactive Plus LC/MS/MS spectrometer was utilized to qualitatively determine the constituents of HS. Chromatographic separation was performed on a Waters U3000 UHPLC system applying an ACQUITY UPLC HSS T3 chromatographic column (100 × 2.1 mm^2^, 1.8 μm, i.d.) at 35°C using a gradient elution at a flow rate of 0.3 mL/min. The mobile phase consisted of 0.1% formic acid-water (A) and acetonitrile (B), and the sample injection volume was set as 10 μL. The gradient program was set as follows: 0–10 min, 0–30% B; 10–25 min, 30–40% B; 25–30 min, 40–50% B; 30–40 min, 50–70% B; 40–45 min, 70–100% B; and 45–60 min, 100% B. All MS experiments were conducted on the Full MS-ddMS2 model with negative and positive scan modes, and the mass scan range was m/z 100–1200. The original raw mass spectra were extracted using Compound Discover 3.2 software, and data analysis was carried out by using the online database, mzCloud, and the self-constructed local database, mzVault (21).

### Myocardial Ischemia/Reperfusion Model and Drug Administration

To establish the myocardial I/R model, male SD rats were anesthetized with inhaled isoflurane (1–2%) throughout the procedure. A tracheal cannula was inserted via rat mouth, with one end being connected with a positive pressure respirator (ALC-V8, Shanghai Alcott Biotech Co., China), which was set at the breathing ratio of 1:1, the frequency of 75/min, and tidal volume of 12 ml/kg. A thoracotomy was performed to expose the heart, and the left anterior descending coronary artery (LADCA) was ligated with a 5/0 silk for 30 min, and was then followed by reperfusion for 120 min (22).

A total of 145 rats were randomly divided into five groups: (1) Sham group (Sham), (2) I/R group (I/R), (3) treatment with 1 g/kg HS in I/R model (HS1 + I/R), (4) treatment with 2 g/kg HS in the I/R model (HS2 + I/R), and (5) treatment with 4 g/kg HS in I/R model (HS4 + I/R). Animals in the Sham group underwent all surgical procedures, except that the silk passing around LADCA was not tied. Rats treated with HS in I/R groups were administered with HS at the dose indicated by gavage once a day for seven consecutive days before the I/R procedure, while the animals in the Sham and I/R groups were administrated normal saline instead in the same way. All indicators were evaluated at 120 min after reperfusion, and rats were euthanized by exposure to an overdose of anesthesia (inhalation of 5% isoflurane). The number of animals used for indicators is detailed in Table 1.

**TABLE 1:** Number of animals included in different experimental groups and various parameters.

Groups	ECG, and hemo-dynamics tests	Evans blue-TTC staining	ELISA for plasma		Proteomic analysis	Western blotting	TEM, IF for ROS	Total
			**HE, TUNEL**	**ELISA for left ventricle**				
Sham	6	6	3	6	3	4	3	31
I/R	7	6	3	6	3	4	3	32
HS1 + I/R	7	6	3	6	0	4	3	29
HS2 + I/R	0	6	3	6	0	4	3	22
HS4 + I/R	6	6	3	6	3	4	3	31
Total	26	30	15	30	9	20	15	145

*The parameters in a column used the same animals from each group for assessment. Sham, Sham group; I/R, I/R 120 min group; HS1 + I/R, pre-treatment with HS at 1 g/kg plus I/R 120 min group; HS2 + I/R, pre-treatment with HS at 2 g/kg plus I/R 120 min group; HS4 + I/R, pre-treatment with HS at 4 g/kg plus I/R 120 min group; ECG, electrocardiograph; TTC, 2, 3, 5-triphenyltetrazolium chloride; TUNEL, terminal deoxynucleotidyl transferase-mediated dUTP nick end labeling; ELISA, enzyme-linked immunosorbent assay; TEM, transmission electron microscope; IF, immunofluorescence; ROS, reactive oxygen species.*

### Electrocardiogram and Hemodynamic Tests

Before the operation and at 120 min after reperfusion, 12-lead electrocardiogram (ECG) was performed and recorded at the rate of 50 mm/s. ST-segment elevation of thoracic lead II at 120 min after reperfusion was analyzed.

At 120 min after reperfusion, cannulation was inserted into the left ventricle through the right carotid artery, which was connected to a bio-function experiment system (BIOPAC MP150). Arterial blood pressure, heart rate (HR), left ventricular systolic pressure (LVSP), left ventricular diastolic pressure (LVDP), left ventricular developed pressure, left ventricular maximum upstroke velocity (+dp/dt max), and left ventricular maximum descent velocity (-dp/dt max) were measured.

### Myocardial Infarct Size Assessment

At 120 min after reperfusion, LADCA was ligated again, and 2 mL of 0.4% Evans blue (Beijing KEHBIO Technology Co. Ltd, China) was administrated through the femoral vein. Hearts were rapidly excised and cut into five sections (1 mm thick), parallel to the atrioventricular groove from the apex cordis to the ligation site. Sections were incubated in a 0.375% solution of 2, 3, 5-triphenyltetrazolium chloride (TTC) (Beijing Solarbio Science Technology Co. Ltd, China) at 37°C for 15 min and then photographed with a stereoscope connected to Digital Sight (DS-5M-U1, Nikon, Nanjing, China). The infarction zone was stained white, the area at risk (AAR) was pink, and the non-infarction area was blue. Myocardial infarct size, AAR, and left ventricle (LV) size were analyzed on each slice by Image-Pro Plus 6.0 (Media Cybernetics, Bethesda, MD, United States) (*n* = 6). The percentage of AAR/LV (%) and infarct size/AAR (%) were calculated, and the values from five slices were averaged and used to score the degree of myocardial infarction (23).

### Myocardial Morphology Evaluation, Immunofluorescence Staining of Terminal Deoxynucleotidyl Transferase-Mediated dUTP Nick End Labeling, and ROS Staining Using Dihydroethidium

At 120 min after reperfusion, rat hearts were removed and fixed in 4% paraformaldehyde. Serial paraffin sections (5 μm) were prepared and stained with HE, and a TUNEL assay was performed using a FITC cell apoptosis detection kit (G1501, Servicebio, Wuhan, China), according to the manufacturer’s instruction (23). In addition, hearts from other rats were excised as above, and frozen sections (8 μm thickness) were prepared. The immunofluorescence staining of ROS was performed using DHE (D7008, Sigma, United States), and the nuclei were labeled with DAPI (G1012, Servicebio, Wuhan, China).

The HE images were observed under an optical microscope (Nikon Eclipse E100, Nikon, Japan) and captured under an imaging system (Nikon DS-U3, Nikon, Japan). Images of IF staining for TUNEL and ROS were observed under an ortho-fluorescent microscope (Nikon Eclipse C1, Nikon, Japan) and captured under an imaging system (Nikon DS-U3, Nikon, Japan). Four fields were selected from the surrounding infarction areas of the left ventricle for each section at ×20 magnification of the objective, and the number of the TUNEL-positive cells in the four fields was counted, and the average was calculated and expressed as cell number per field.

### Enzyme-Linked Immunosorbent Assay

Enzyme-linked immunosorbent assay (ELISA) was performed using a specific kit indicated by a microplate reader (RT-6100, Rayto, Shenzhen, China), respectively, to determine the content of creatine kinase-MB (CK-MB), troponin T (cTnT), lactic dehydrogenase (LDH), alanine transaminase (ALT), aspartate transaminase (AST), ROS, NO, RNS, IL-1β, IL-18, TNF-α, and IL-6 in plasma (Dogesce Biotech, Beijing, China), and 8-OHdG, malonaldehyde (MDA), and NAD^+^ in the left ventricle (Dogesce Biotech, Beijing, China). Likewise, the activities of caspase-1, MnSOD, and mitochondrial Complexes I, II, IV, and V were also detected using the ELISA kit according to the manufacturer’s instructions (24).

### Proteomics Analysis

After observation for 120 min, tissue proteins surrounding the left ventricular infarction from three groups (Sham, I/R, HS4 + I/R) were extracted and quantified using the BCA Protein Assay Kit (Thermo Fisher Scientific, Waltham, MA, United States). Then the proteins were labeled with tandem mass tags (TMT), and the peptides were reseparated using an 1100 HPLC System (Agilent) with an Agilent Zorbax Extend RP column. The separated peptides were lyophilized for further MS detection using a Q Exactive Mass Spectrometer (Thermo Fisher, United States). The mass spectrometry resolution was 60,000 FWHM at *m/z* 400. Spectra were collected using full ion scan mode over the mass-to-charge *(m/z)* ratio range of 300–1,600 au in positive mode, and the strongest 10 pieces of the MS/MS scan were analyzed in the linear ion trap using collision-induced dissociation (collision energy = 30 eV). Proteome Discover (v. 2.2) was used to process RAW instrument files against the Mus musculus UniProt protein database. The MS/MS data were collected and searched against the Oryctolagus cuniculus database using the Sequest algorithms. Search results were filtered to a 1% protein false discovery rate using Percolator. Only peptides with a q score < 0.05 were accepted, and at least 1 unique peptide was required for quantification. The data were corrected for systematic differences resulting from labeling efficiency by normalizing the reporter ion totals. Three biological replicates were analyzed, and each sample was adjusted to the same intensity of pooled internal standard within each TMT experiment. Only protein groups without any missing values in all samples were selected for Student’s *t*-test (24).

### Western Blotting Assay

The Western blotting assay was performed to detect the expression of OXPHOS, MnSOD (acetyl K68 and Acy-MnSOD), MnSOD, NLRP3, ASC, and GSDMD. Myocardial tissues were taken from the left ventricle around the infarction zone, and total proteins were extracted using a protein extraction kit (Applygen Technologies, Beijing, China) and mixed with a 5 × electrophoresis sample buffer. After electrophoresis, the separated proteins were transferred to a polyvinylidene difluoride membrane and then blocked with 5% non-fat dry milk. The membrane with target proteins was incubated overnight at 4° with antibodies against OXPHOS (ab110413, abcam, Cambridge, MA, United States), Acy-MnSOD (ab137037, abcam, Cambridge, MA, United States), MnSOD (GB111875, Servicebio, Wuhan, China), NLRP3 (ab263899, abcam, United States), ASC (ab180799, abcam, Cambridge, MA, United States), and GSDMD (39754S, Cell Signaling Technology, Boston, MA, United States). Blotted antibodies were visualized by using an HRP-conjugated secondary antibody (7074S, Cell Signaling Technology, Boston, MA, United States) and ECL detection system (P1010, Applygen Technologies, Beijing, China). Densitometric analyses of blots were performed using the Quantity One image analyzer software (Bio-Rad, Richmond, CA, United States). The result of each band was expressed as relative optical density and compared with internal controls of GAPDH (22).

### Myocardial Ultrastructure Examination

An approximately 1 mm^3^ fresh myocardial tissue block around the infarction zone was taken from the left ventricle. Tissues were fixed with 3% glutaraldehyde and post-fixed with 1% osmium tetraoxide. The specimens were processed as routine for ultrathin sections. Sections were stained with uranium acetate and lead citrate, and ultrastructural changes were then evaluated by using a transmission electron microscope (TEM) (Hitachi TEM system, Tokyo, Japan) (24).

### Statistical Analysis

All data were expressed as mean ± SD values. The histograms of parameters were plotted with GraphPad Prism 6 software, and statistical analysis was carried out using one-way ANOVA, followed by the Bonferroni test for multiple comparisons. A probability less than 0.05 was considered to be statistically significant.

## Results

### The Major Chemical Constituents in Herba Siegesbeckiae Aqueous Extract

**FIGURE 1:**
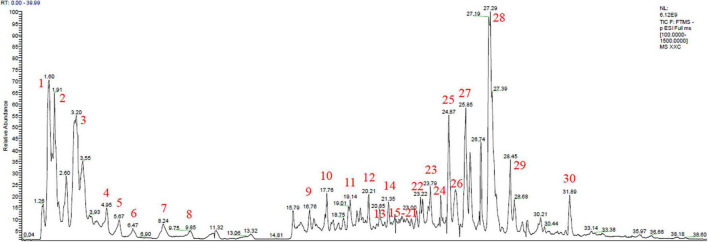
Total ion chromatogram (TIC) scan of Herba Siegesbeckiae (HS) in negative mode.

On the basis of the total ion chromatogram (TIC) in negative mode plotted in Figure 1, a total of 22 known chemical constituents and eight unreported sulfonic acid derivatives were identified from the extract of HS, which were mainly divided into three clusters, including flavonoids, diterpenoids, and organic acids, detailed in Table 2. It is noteworthy that compounds **23–30** with the retention time (RT) of 23.79, 24.39, 24.87, 25.25, 25.85, 27.29, 27.53, and 31.89 min, respectively, were determined as sulfonic acid derivatives according to its characteristic chemical formula of C_20/22_H_30/32/34/36_O_7/8_S, which were unreported in HS and showed the similar MS^2^ with kaurenoid-type diterpenoids (Supplementary Figure 1). In addition, compounds **1, 3, 5, 6, 10, 12**, and **22** with RT at 1.60, 3.20, 5.67, 6.47, 17.76, and 20.21 min, respectively, were elucidated as organic acids, including quinic acid **(1)**, citric acid **(3)**, 3′-guanylic acid **(5)**, 5′-guanylic acid **(6)**, ZINC100070393 **(10)**, 3,4-dihydroxyphenylethanol **(12)**, and Azelaic acid **(22)**. Compounds **11 – 21** except 12, with RT of 19.14, 20.85, 21.35, 21.82, 21.82, 21.85, 21.89, 22.15, 22.85, and 22.95 min, respectively, were recognized as flavonoid glycosides, such as Vicenin II **(11)**, Orientin **(13)**, Vitexin **(14)**, Hyperoside **(15)**, Quercetin **(16)**, Cynaroside **(17)**, Luteolin 7-glucuronide **(18)**, Genistin **(19)**, Apigenin-7-*O*-β-D-glucoside **(20)**, and Apigenin 7-*O*-glucuronide **(21)**. The TIC in positive and negative mode, UV absorbance at 230 nm, and accumulated UV absorbance are presented in Supplementary Figure 2.

**TABLE 2:** Information of 30 components of Herba Siegesbeckiae (HS) aqueous extract determined by LC-MS (*, unreported compound in HS).

No.	Molecular formula	[M-H]^–^/[M + H]^+^	t_*R*_ (min)	Identification	Classification
1	C_7_H_12_O_6_	191.0558/–	1.60	Quinic acid	Organic acid
2	C_7_H_13_NO_2_	–/144.1017	1.91	Stachydrine	Alkaloid
3	C_6_H_8_O_7_	191.0195/193.0341	3.20	Citric acid	Organic acid
4	C_24_H_42_O_21_	665.2137/–	4.95	Stachyose	Saccharide
5	C_10_H_14_O_7_N_5_P	346.0553/348.0699	5.67	3′-guanylic acid	Organic acid
6	C_10_H_14_N_5_O_8_P	362.0503/364.0648	6.47	5′-Guanylic acid	Organic acid
7	C_10_H_12_O_7_N_5_P	344.0395/346.0543	8.24	Guanosine 3′,5′- cyclic monophosphate	Nucleotide
8	C_30_H_52_O_26_	827.2663/–	9.85	Maltopentaose	Saccharide
9	C_13_H_16_O_9_	153.0555/–	16.76	3,4-Dihydroxyphenylethanol	Aromatics
10	C_16_H_18_O_9_	353.0872/355.1017	17.76	ZINC100070393	Organic acid
11	C_27_H_30_O_15_	593.1505/–	19.14	Vicenin II	Flavonoid
12	C_25_H_24_O_12_	515.1185/517.1335	20.21	1,3-Dicaffeoylquinic acid	Organic acid
13	C_21_H_20_O_11_	447.0927/449.1083	20.85	Orientin	Flavonoid
14	C_21_H_20_O_10_	–/443.1126	21.35	Vitexin	Flavonoid
15	C_21_H_20_O_12_	463.0876/–	21.82	Hyperoside	Flavonoid
16	C_15_H_10_O_7_	–/303.0495	21.82	Quercetin	Flavonoid
17	C_21_H_20_O_11_	–/249.1073	21.85	Cynaroside	Flavonoid
18	C_21_H_18_O_12_	–/463.0866	21.89	Luteolin 7-glucuronide	Flavonoid
19	C_21_H_20_O_10_	–/433.1124	22.15	Genistin	Flavonoid
20	C_21_H_20_O_10_	–/443.1126	22.85	Apigenin-7-*O*-β-D-glucoside	Flavonoid
21	C_21_H_18_O_11_	–/447.0917	22.95	Apigenin-7-*O*-glucuronide	Flavonoid
22	C_9_H_16_O_4_	187.0972/–	23.23	Azelaic acid	Organic acid
23*	C_20_H_34_O_7_S	417.1945/–	23.79	Sulfonic acid derivatives	Diterpene
24*	C_22_H_34_O_8_S	457.1895/459.2093	24.39	Sulfonic acid derivatives	Diterpene
25*	C_20_H_32_O_7_S	415.1788/417.1934	24.87	Sulfonic acid derivatives	Diterpene
26*	C_20_H_30_O_7_S	413.1633/–	25.25	Sulfonic acid derivatives	Diterpene
27*	C_22_H_36_O_8_S	459.2050/–	25.85	Sulfonic acid derivatives	Diterpene
28*	C_22_H_34_O_8_S	457.1895/459.2032	27.29	Sulfonic acid derivatives	Diterpene
29*	C_22_H_32_O_8_S	455.1739/457.1883	27.53	Sulfonic acid derivatives	Diterpene
30*	C_22_H_34_O_7_S	441.1944/443.2092	31.89	Sulfonic acid derivatives	Diterpene

### Herba Siegesbeckiae Decreased Ischemia/Reperfusion-Induced Myocardial Infarct Size

**FIGURE 2:**
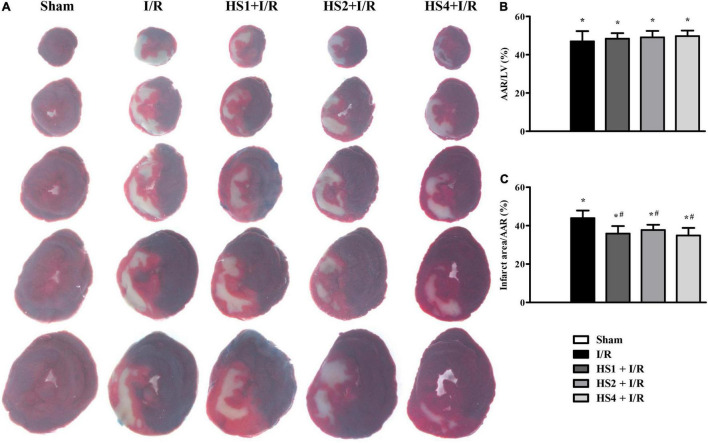
Effect of Herba Siegesbeckiae (HS) on Ischemia/Reperfusion (I/R)-induced myocardial infarct size in rats. **(A)** Representative myocardial tissue slices stained by Evans blue-TTC in Sham, I/R, HS1 + I/R, HS2 + I/R, and HS4 + I/R groups. **(B)** Quantitative analysis of AAR/LV in various groups (*n* = 6). **(C)** Quantitative analysis of infarct area/AAR in various groups (*n* = 6). *P < 0.05 vs. Sham group, ^#^*P* < 0.05 vs. I/R group.

To investigate the cardioprotective effect of HS, myocardial infarct of various groups was assessed by performing Evans blue-TTC staining at 120 min after reperfusion. The representative images in different groups are illustrated in Figure 2A, wherein the blue area represents a non-ischemic region, the red area represents an ischemic region, and the white area represents the infarction region. Apparently, no ischemia and infarct were observed in the myocardial tissue slices obtained from the Sham group. On the contrary, noticeable ischemia and infarct areas were observed in the I/R groups compared to the Sham group, but such myocardial infarct was significantly diminished in the myocardial tissues extracted from the groups receiving pre-treatment with HS.

The quantitative analysis of AAR/LV and infarct size/AAR is shown in Figures 2B,C, respectively. Compared with the Sham group, AAR/LV and infarct size/AAR increased markedly in the I/R group and I/R administrated with HS groups (*P < 0.05 vs. Sham). There was no significant difference in AAR/LV between the I/R group and HS + I/R groups (*P* > 0.05). However, a noticeable increase in the infarct size/LV in the I/R group was significantly alleviated by treatment with HS at the three doses tested (^#^*P* < 0.05 vs. I/R), suggesting that HS exerts therapeutic effects on I/R-evoked myocardial infarction.

### Herba Siegesbeckiae Reduced ST-Segment Elevation and Ameliorated Cardiac Function Induced by Ischemia/Reperfusion

**FIGURE 3:**
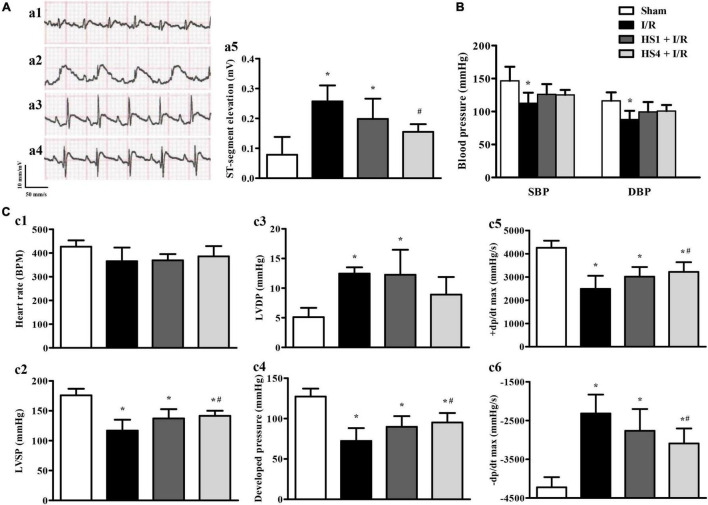
Effect of Herba Siegesbeckiae (HS) on ST-segment, blood pressure, and cardiac function induced by Ischemia/Reperfusion (I/R). **(A)** Representative ECG of thoracic lead II in Sham (a1), I/R (a2), HS1 + I/R (a3), and HS4 + I/R (a4) groups, and quantitative analysis of ST-segment elevation among the four groups at 120 min after reperfusion (a5). **(B)** Quantitative analysis of systolic blood pressure (SBP) and diastolic blood pressure (DBP) in Sham (*n* = 6), I/R (*n* = 7), HS1 + I/R (*n* = 7), and HS4 + I/R (*n* = 6) groups. **(C)** Quantitative analysis of heart rate (HR, c1), left ventricular systolic pressure (LVSP, c2), left ventricular diastolic pressure (LVDP, c3), left ventricular developed pressure (c4), left ventricular maximum upstroke velocity (+ dp/dt max, c5), and left ventricular maximum descent velocity (-dp/dt max, c6) in Sham (*n* = 6), I/R (*n* = 7), HS1 + I/R (*n* = 7), and HS4 + I/R (*n* = 6) groups. *P < 0.05 vs. Sham group, ^#^*P* < 0.05 vs. I/R group.

To investigate the effect of HS on ECG, blood pressure, and cardiac function after I/R, the 12-lead ECG and hemodynamics tests were conducted. As shown in Figure 3A, the ST-segment was significantly elevated after I/R (a2) compared to that observed in the Sham (a1) group, while HS at dosages of 1 g/kg (a3) and 4 g/kg (a4) obviously reduced the elevation of ST-segment induced by I/R challenge, which was further confirmed by the quantitative results presented in Figure 3A.

The effects of HS on systolic blood pressure (SBP) and diastolic blood pressure (DBP) are shown in Figure 3B. Compared with the Sham group, I/R elicited a remarkable decline in SBP and DBP, and HS pre-treatment reversed the decrease of SBP and DBP to a certain extent, but there was no statistical significance between the HS groups and I/R group.

Figure 3C displays the changes in HR (c1), LVSP (c2), LVDP (c3), developed pressure (c4), +dp/dt max (c5), and -dp/dt max (c6) in various groups. Notably, there is no apparent difference in HR among the four groups. While in comparison with the Sham group, I/R caused a significant decline in LVSP, left ventricular developed pressure, and +dp/dt max, and an increment in LVDP and -dp/dt max, implying an impairment of heart function. Obviously, these impairments were ameliorated by pre-treatment with HS at the dose of 4 g/kg, except for that in LVDP.

### Herba Siegesbeckiae Retained Myocardial Morphology, Alleviated Myocardial Apoptosis, and Myocardial Injury After Ischemia/Reperfusion

**FIGURE 4:**
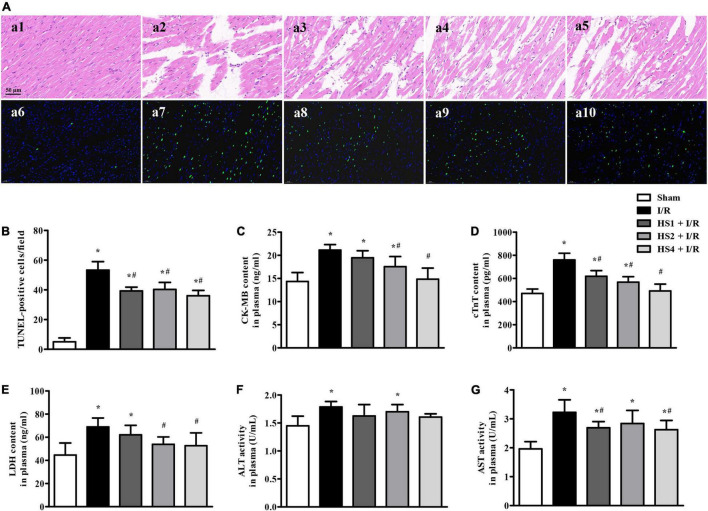
Effect of Herba Siegesbeckiae (HS) on myocardial histology, apoptosis, and injury after Ischemia/Reperfusion (I/R). **(A)** Representative HE-stained (a1–a5) and TUNEL staining (a6–a10) photographs in Sham (a1,a6), I/R (a2,a7), HS1 + I/R (a3,a8), HS2 + I/R (a4,a9), and HS4 + I/R (a5,a10) groups. Bar = 50 μm. **(B)** The quantitative analysis of TUNEL-positive cells/field in different groups (*n* = 3). **(C–G)** The plasma contents of CK-MB **(C)**, cTnT **(D)**, LDH **(E)**, and activities of ALT **(F)** and AST **(G)** in different groups (*n* = 9). *P < 0.05 vs. Sham group, ^#^*P* < 0.05 vs. I/R group.

To evaluate the effect of HS on I/R-induced myocardial structure and apoptosis, HE staining and TUNEL staining were performed, and the results are presented in Figure 4A. The upper panels (a1–a5) are representative HE micrographs captured under ×40 magnification of the objective, depicting that I/R challenge (a2) elicited a distinct morphological injury, such as myocardial fiber disarrangement, disruption, and myocardial tissue edema compared with Sham group (a1). Noticeably, HS administration at the dose of 1 g/kg (a3), 2 g/kg (a4), and 4 g/kg (a5) significantly preserved the myocardium structure after I/R.

The lower panels of Figure 4A (a6–a10) display the representative images of TUNEL staining, wherein nuclei are stained blue and TUNEL-positive cells green. Obviously, no or few TUNEL-positive cells were seen in the Sham group (a6). In contrast, a large number of TUNEL-positive cells were observed in the I/R group (a7), which were evidently reduced by HS. The quantitative analysis of TUNEL-positive cells/field (Figure 4B) confirmed the above results.

In order to investigate the effect of HS on cardiac lesion enzymes, the plasma contents of CK-MB, cTnT, and LDH, and the activities of ALT and AST were detected. As shown in Figures 4C–G, I/R evoked an obvious increase of CK-MB, cTnT, LDH, ALT, and AST in comparison with the Sham group (*P < 0.05 vs. Sham). However, the HS treatment, especially at the doses of 4 g/kg, remarkably inhibited the elevation of the above enzymes caused by I/R, implying that pre-treatment with HS apparently alleviates I/R-induced myocardial injury.

### Proteomics Analyses Revealed That Ischemia/Reperfusion Evoked Oxidative Stress and Inflammation

**FIGURE 5:**
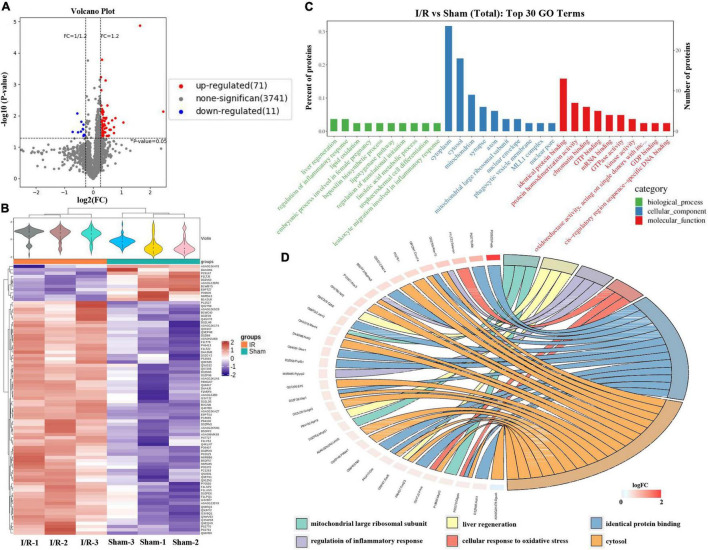
Quantitative proteomics analysis of Ischemia/Reperfusion (I/R) group compared with Sham group. **(A)** The volcano plot of differentially expressed proteins (DEPs) identified between the I/R group and the Sham group. Red dots represent upregulated DEPs, blue dots represent downregulated DEPs, and gray dots represent no significant proteins. **(B)** The clustering heat map between I/R and Sham group. **(C)** The top 30 significantly enriched GO terms for the DEPs identified between the I/R group and Sham group using biological process (BP), cellular component (CC), and molecular function (MF). **(D)** The chord diagram of GO enrichment analysis, displaying the first 6 GO terms are cytosol, identical protein binding, cellular response to oxidative stress, regulation of inflammatory response, liver regeneration, and mitochondrial large ribosomal subunit.

A total of 4,279 proteins were identified by LC-MS/MS, among which 3,823 trusted proteins were screened out, according to the standard of Score Sequest HT > 0 and unique peptide ≥ 1 without the blank value. The Volcano Plot and the clustering heat map of I/R vs. Sham are shown in Figures 5A,B, displaying that a total of 82 differentially expressed proteins (DEPs) were identified between the I/R group and the Sham group, 71 of which were upregulated, while 11 were downregulated. The top 30 remarkable GO enrichment terms between I/R and Sham groups are demonstrated in Figure 5C. The number of DEPs in each term greater than 3 and less than 5 were selected, and the first 6 terms were sorted from large to small according to the corresponding −log10 *P*-value of each item to obtain the GO enrichment analysis chord diagram. As shown in Figure 5D, the first 6 terms are cytosol, identical protein binding, cellular response to oxidative stress, regulation of inflammatory response, liver regeneration, and mitochondrial large ribosomal subunit.

### Herba Siegesbeckiae Regulated Mitochondrion and the Expression of Adgb, Cbr1, Decr1, Eif5, Uchl5, Lmo7, Bdh1, Ckmt2, COX7A, and RT1-CE1 After Ischemia/Reperfusion

**FIGURE 6:**
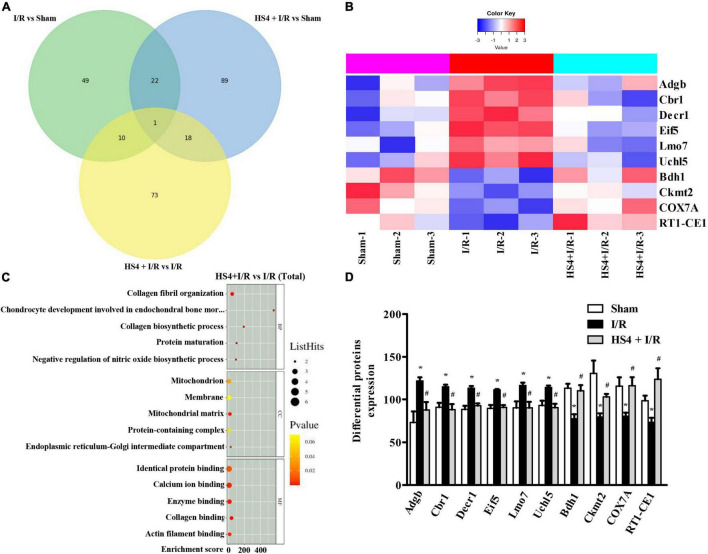
Quantitative proteomics analysis of HS4 + I/R group compared with I/R. **(A)** The Venn diagram of differentially expressed proteins (DEPs) among the three groups of Sham, I/R, and HS4 + I/R. **(B)** The bubble diagram of the top 15 enriched GO terms identified between HS4 + I/R group and I/R group using BP, CC, and MF. **(C,D)** The heat map and quantitative statistics of DEPs in Sham group, I/R group, and HS4 + I/R group.

As shown in the Venn diagram in Figure 6A, 102 DEPs were observed between HS4 + I/R and I/R groups, and the bubble diagram of GO enrichment analysis for the 102 DEPs is demonstrated in Figure 6B. Cellular component (CC) analysis showed that the DEPs between HS4 + I/R and I/R groups were mainly enriched in “mitochondrion”. Additionally, 11 DEPs were detected among the three groups, that is, Sham, I/R, and HS4 + I/R. Further analysis of the 11 DEPs among the three groups displayed that except for Junb, the remaining 10 proteins, Adgb, Cbr1, Decr1, Eif5, Uchl5, Lmo7, Bdh1, Ckmt2, COX7A, and RT1-CE1, exhibited opposite pattern in the HS4 + I/R group compared to that observed in the I/R group. The heat map and statistical analysis of the 10 DEPs are demonstrated in Figures 6C,D. The molecular functions, biological processes, and the related pathways of the 10 DEPs are detailed in Table 3.

**TABLE 3:** Molecular functions, biological processes, and related pathways of the 10 differentially expressed proteins (DEPs) among the three groups of Sham, I/R, and HS + I/R.

DEPs	Molecular functions	Biological process and related pathways
Adgb	Oxygen carrier and binding	NO metabolism, peroxidase activity
Cbr1	Oxidoreductase activity	Tetrahydrobiopterin biosynthetic process, ROS biosynthetic process, NO metabolism
Decr1	Dodecenoyl-CoA delta-isomerase activity, acyl-CoA dehydrogenase activity, oxidoreductase activity	Fatty acid beta-oxidation, carnitine metabolic process, oxidative stress and lipid peroxidation
Eif5	Translation initiation factor binding/activity	Eukaryotic translation initiation, GTP hydrolysis, response to stress
Uchl5	Ubiquitin carboxyl-terminal hydrolase activity	Oxygen-dependent proline hydroxylation of HIF-α, IL-1 signaling, neutrophil degranulation
Lmo7	Muscle alpha-actinin binding, actin binding, metal ion binding	Adherens junction, regulation of actin cytoskeleton
Bdh1	Hydroxymethylglutaryl-CoA lyase activity, 3-hydroxybutyrate dehydrogenase activity	Ketone body metabolism, fatty-acid beta-oxidation, acyl-CoA metabolic process, response to stress
Ckmt2	Porin activity, creatine kinase activity	Couples ATP production to phosphocreatine, calcium signaling, NOD-like receptor signaling pathway
COX7A	Cytochrome-c oxidase activity	Mitochondrial electron transport, cytochrome c to oxygen, ubiquinol to cytochrome c, oxidative phosphorylation
RT1-CE1	MHC class I protein complex binding	Antigen processing and presentation, T cell receptor signaling pathway, cell adhesion molecules, adaptive immune system

*NO, nitric oxide; HIF-α, hypoxia-inducible factor-α*

### Herba Siegesbeckiae Preserved Myocardial Ultrastructure and Restored Mitochondrial Electron Transport Chain Complex Function Following Exposure to Ischemia/Reperfusion

**FIGURE 7:**
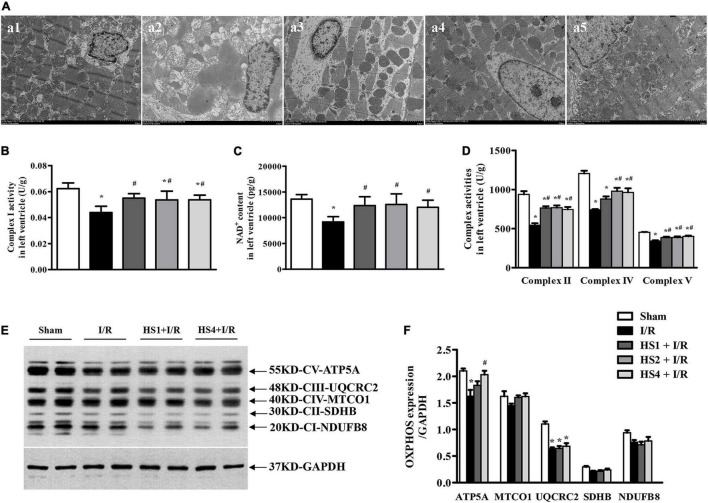
Effect of Herba Siegesbeckiae (HS) on myocardial ultrastructure and mitochondrial ETC function. **(A)** Representative images of myocardial ultrastructure acquired at × 2.5 K magnification in Sham (a1), I/R (a2), HS1 + I/R (a3), HS2 + I/R (a4), and HS4 + I/R (a5) groups. **(B,C)** The Complex I activity **(B)** and NAD^+^ content (C) in left ventricle in various groups (*n* = 6). **(D)** The activities of Complexes II, IV, and V in the left ventricle in various groups (*n* = 6). **(E)** The representative image of OXPHOS expression. **(F)** The quantitative analysis of OXPHOS expression (*n* = 4). *P < 0.05 vs. Sham group, ^#^*P* < 0.05 vs. I/R group.

To investigate the effect of HS on mitochondrion after I/R, myocardial ultrastructure was observed by TEM. The representative myocardial electron micrographs captured at 2.5 K magnification are demonstrated in Figure 7A. As noticed, the myocardium in Sham group (a1) presented the characteristics of normal structure with regularly arranged and densely packed myofibrils and mitochondria, and clearly visible mitochondrial cristae. In comparison with the Sham group, the I/R challenge evoked a dramatic myocardial ultrastructure injury, as indicated by the rupture of myocardial fibers, tissue edema, mitochondrial swelling, and disrupted cristae (a2). However, the I/R-elicited remarkable alterations in the myocardial ultrastructure were apparently alleviated in the groups receiving HS, especially at the dose of 4 g/kg (a5).

To further explore the effect of HS on the mitochondrial function, the activities of ETC complexes and the expression of OXPHOS in the left ventricle were detected. Notably, the activities of Complexes I (Figure 7B), II (Figure 7D), IV (Figure 7D), and V (Figure 7D) were prominently inhibited by I/R compared to those observed in the Sham group, while HS significantly restored the activities of the above complexes. As a Complex I catalyzed product, the variation trend of NAD^+^ content in various groups (Figure 7C) was parallel with the variation pattern of Complex I activity.

Furthermore, the expression of OXPHOS was detected, as shown in Figure 7E. The representative image demonstrates NADH dehydrogenase beta subcomplex subunit 8 of Complex II (NDUFB8), succinate dehydrogenase subunit B of Complex I (SDHB), cytochrome c oxidase subunit 1 of Complex IV (MTCO1), cytochrome b-c1 complex subunit 2 of Complex III (UQCRC2), and ATP synthase subunit alpha of Complex V (ATP5A). It is obvious from the statistical analysis of Figure 7F that the expression of ATP5A and UQCRC2 was significantly decreased by I/R, while HS at the dose of 4 mg/kg could reverse the reduction of ATP5A induced by I/R. In addition, the expression of UQCRC2, SDHB, and NDUFB8 was also examined by proteomic analysis (Supplementary Figure 4), and their variation trends were similar to the Western blotting results.

### Herba Siegesbeckiae Suppressed Ischemia/Reperfusion-Elicited Increase of ROS, RNS, MDA, and 8-OHdG, Restrained the Acetylation of MnSOD, and Recovered the Activity of MnSOD

**FIGURE 8:**
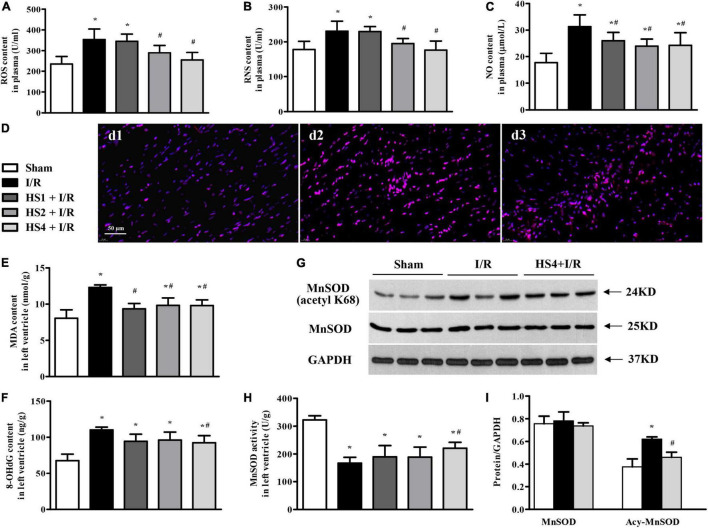
Effect of Herba Siegesbeckiae (HS) on Ischemia/Reperfusion (I/R)-elicited oxidative stress. **(A–C)** The plasma contents of ROS **(A)**, RNS **(B)**, and NO **(C)** in different groups (*n* = 9). **(D)** Representative ROS immunofluorescence photographs stained by DHE in Sham (d1), I/R (d2), and HS4 + I/R (d3) groups. Bar = 50 μm. **(E,F)** The contents of MDA **(E)** and 8-OHdG **(F)** in the left ventricle in various groups (*n* = 6). **(G,H)** The representative protein expression and quantitative analysis of MnSOD and Acy-MnSOD in left ventricle (*n* = 3). **(I)** The activity of MnSOD in the left ventricle in different groups (*n* = 6). *P < 0.05 vs. Sham group, ^#^*P* < 0.05 vs. I/R group.

To address the oxidative stress in various conditions, ROS, RNS, and NO contents in plasma, and MDA and 8-OHdG contents in the left ventricle were determined by ELISA. Of notice, the I/R challenge induced a remarkable increase in the contents of ROS (Figure 8A), RNS (Figure 8B), NO (Figure 8C), MDA (Figure 8E), and 8-OHdG (Figure 8F) compared with the Sham group. However, the above I/R-evoked indicators of oxidative stress were prominently attenuated by HS, particularly at the dose of 4 g/kg.

Furthermore, ROS in the left ventricle was evaluated by immunofluorescence staining with DHE. The representative images of ROS staining, manifesting as a pink zone, are displayed in Figure 8D. Apparently, there was little ROS-positive staining in the Sham group (d1), while a noticeable elevation in ROS-positive staining was observed in the surrounding infarction areas of myocardial tissues extracted from the I/R group (d2). However, the prominent increase of ROS-positive staining is obviously reversed by HS at the dose of 4 g/kg (d3).

To explore the antioxidant mechanism of HS, the expression and acetylation of MnSOD were examined. As shown in Figure 8G, there was no significant alteration in MnSOD among Sham, I/R, and HS4 + I/R groups, while the expression of Acy-MnSOD was obviously increased by I/R compared with the Sham group and decreased after HS administration. The quantitative analysis (Figure 8H) confirmed the results of protein expression bands. Additionally, MnSOD activity in the left ventricle (Figure 8I) exhibited an opposite pattern to that of Acy-MnSOD expression. These data highlight the involvement of MnSOD in the antioxidant effect of HS.

### Herba Siegesbeckiae Reversed Ischemia/Reperfusion-Induced Elevation of NLRP3 Inflammasome and Inhibited Release of Inflammatory Factors and Pyroptosis

**FIGURE 9:**
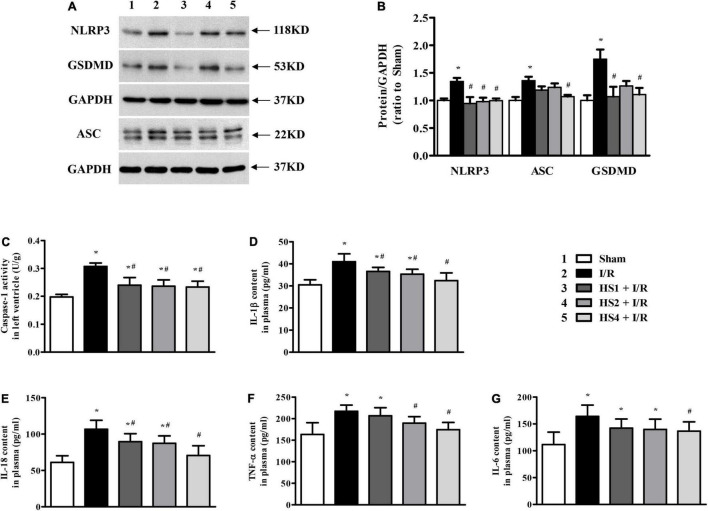
Effect of Herba Siegesbeckiae (HS) on NLRP3 inflammasome and inflammatory response. **(A)** Representative Western blotting bands of NLRP3, ASC, and GSDMD in various groups. **(B)** The semi-quantitative analysis of NLRP3, ASC, and GSDMD in various groups (*n* = 4). **(C)** The caspase-1 activity in left ventricle in different groups (*n* = 6). **(D–G)** The plasma contents of IL-1β **(D)**, IL-18 **(E)**, TNF-α **(F)**, and IL-6 **(G)** in different groups (*n* = 9). *P < 0.05 vs. Sham group, ^#^*P* < 0.05 vs. I/R group.

To further gain insight into the effect of HS on I/R-induced inflammation, we focused on the NLRP3 inflammasome. As shown in Figure 9A, the expression of NLRP3 and ASC was apparently augmented in the I/R group compared with the Sham group. On the contrary, the obvious elevations in the NLRP3 and ASC expression were significantly suppressed by HS, which were confirmed by the quantitative analysis results (Figure 9B). As the effector of the NLRP3 inflammasome, the activity of caspase-1 was also elevated by I/R while was diminished by HS (Figure 9C), which was in line with the expression pattern of NLRP3 and ASC.

As the downstream targets of caspase-1, IL-1β, and IL-18 contents in the plasma were examined, and GSDMD protein expression was assessed. Consistent with the alteration of caspase-1, the plasma levels of IL-1β (Figure 9D) and IL-18 (Figure 9E) were obviously increased after the I/R challenge, while such augmentation was remarkably reversed by HS. Likewise, the expression of GSDMD exhibited a comparable pattern to that of caspase-1 (Figures 9A,B), implying the ameliorating effect of HS on pyroptosis evoked by I/R. Impressively, as the key factors to trigger inflammatory cascades, the increase in TNF-α (Figure 9F) and IL-6 (Figure 9G) induced by I/R was apparently restrained by HS, especially at the dose of 4 g/kg.

## Discussion

The present study first demonstrated that pre-treatment with HS aqueous extract notably ameliorated cardiac I/R injury, mainly manifesting as a reduction in the infarct size, apoptotic cells, and cardiac lesion enzymes, decline of ST-segment elevation, improvement of cardiac function, and preservation of myocardial morphology. Quantitative proteomics analysis revealed that the cardioprotective effect of HS on cardiac I/R injury depended on the restoration of the function of mitochondrial ETC complexes and the inhibition of oxidative stress and inflammatory response. The potential mechanism is closely related to the downregulation of Adgb, Cbr1, Decr1, Eif5, Uchl5, and Lmo7 expression, and the upregulation of Bdh1, Ckmt2, COX7A, and RT1-CE1 expression by HS.

Herba Siegesbeckiae, known as “Xi Xian Cao” in China, has been considered to be an effective traditional medicine for treating rheumatoid arthritis, improving joint motion, and relieving pain symptoms (25–27). Recent studies have found that HS ethanol extract shows a cardiovascular protective effect, which could reduce ST-segment elevation after acute myocardial ischemia, reverse pressure overload-induced myocardial remodeling, and alleviate doxorubicin-induced myocardial injury (20). However, the role of HS aqueous extract has not been reported. The present study is the first to confirm the cardioprotective role of HS aqueous extract against cardiac I/R injury. Specifically, administering HS for 7 days prior to I/R markedly attenuated myocardial infarction and apoptosis, reduced ST-segment elevation, and alleviated cardiac dysfunction induced by I/R. The 1, 2, and 4 g/kg dosages of HS used in the present study were 0.5, 1, and 2 times the clinical equivalent dose, respectively. From the present results, the cardioprotective effect of HS at the dose of 4 g/kg seemed to be superior compared to the other two doses, but there was no statistical significance among the three doses. Additionally, 7 days of HS administration at a 4 g/kg dose had no effect on body weight, cardiac function, and heart rate of normal rats without I/R exposure (Supplementary Figure 3). It is necessary to increase the dose of HS in further studies to clarify the optimal dose of HS.

Herba Siegesbeckiae harbors a variety of chemical constituents that exhibit various pharmacological activities, which provide scientific evidence for its clinical application. According to the literature, HS ethanol extracts have been well-studied and separated mainly into five categories: sesquiterpenoids, diterpenoids, flavonoid aglycones, organic acids, and miscellaneous (20, 28). Considering that HS is mainly extracted by water in a clinical setting, the present study focused on the role of HS aqueous extract. Different from flavonoid aglycone and diterpenoids isolated from HS ethanol extract, a large number of flavonoid glycosides and potential sulfonic acid derivatives of diterpenoids were found in the HS aqueous extract, as demonstrated by LC-MS analysis (Figure 1). It is well-known that flavonoids are important oxygen-containing heterocyclic natural products, with antioxidant and anti-inflammatory activities (29), which may be partly responsible for the potential role of HS aqueous extract against myocardial I/R injury. Furthermore, flavonoids isolated from HS aqueous extracts in the present study, such as orientin (30), vitexin (31), hyperoside (32), quercetin (33), cynaroside (34), and genistin (35), have been demonstrated to be cardioprotective during I/R, possibly through a mechanism of regulating mitochondrial function. In addition to flavonoids, other components of HS aqueous extracts, like stachydrine and 3,4-dihydroxyphenylethanol, have also been reported to protect the heart in the models of pressure overload-induced cardiac hypertrophy (36), ISO-evoked heart failure (37), and doxorubicin-induced cardiotoxicity (38). Impressively, the sulfonated diterpenoid derivatives of compounds **23–30** were first reported, and their pharmacological activities and exact chemical structures need to be further elucidated.

A prominent effect of HS aqueous extract observed in the present study is to inhibit oxidative stress via its antioxidant activity, that is, restraining I/R-induced acetylation of MnSOD and improving the activity of MnSOD, thus reducing the levels of ROS, RNS, NO, MDA, and 8-OHdG after I/R. The antioxidant capacity of HS helps to alleviate myocardial apoptosis caused by I/R.

Quantitative proteomic analysis suggested that the downregulation of Adgb, Cbr1, Decr1, and Eif5 by HS may be responsible for the inhibitory effect of HS on oxidative stress. Specifically, Adgb (androglobin) was first identified and termed by Hoogewijs due to its predominant expression in the mammalian testis, and has been reported to be involved in NO metabolism, peroxidase activity, and signal transduction in addition to the basic function of oxygen transport and storage (39, 40). However, its role in the myocardium has not been explored. The present study is the first to report that the expression of Adgb in the myocardium was upregulated by I/R but reversed by HS, which may partly account for the reduction of NO and RNS by HS after the I/R challenge. Cbr1 belongs to NADPH-dependent reductase that participates in a variety of biosynthesis processes, including tetrahydrobiopterin, superoxide, and NO metabolism (41). Previous studies have confirmed the involvement of Cbr1 in neuron and hepatic I/R-induced oxidative stress (42, 43), while this study implies that Cbr1 also took part in cardiac I/R injury. Decr1 is an auxiliary enzyme of fatty acid beta-oxidation located in mitochondria and has been reported to participate in lipid modulation and mitochondrial oxidative stress in prostate cancer (44). The alteration of Decr1 in the present study supports the contribution of Decr1 to cardiac I/R injury-induced oxidative stress. Eif5 promotes the initiation of eukaryotic translation, leading to GTP hydrolysis and release of eIF-2 and the guanine nucleotide (45). The modulation of Eif5 by HS in I/R suggests that HS can inhibit the stress state caused by I/R, but which proteins are mediated by Eif5 and their roles in I/R need further investigation.

Another significant role of HS aqueous extract identified in this study is to suppress cardiac I/R-evoked inflammatory response, especially NLRP3 inflammasome. The results indicated that HS remarkably downregulated NLRP3, ASC, and GSDMD expression and lowered caspase-1 activity, resulting in the reduction of IL-1β and IL-18. It has been validated that NLRP3 inflammasome can be activated by ROS under I/R condition (46); therefore, the restrain of ROS by HS may partly contribute to the inhibition of NLRP3 inflammasome.

Proteomic analysis results implied that the regulation of HS on Uchl5 and Lmo7 may contribute to the potential role of HS in suppressing I/R-induced inflammation. Uchl5, a deubiquitin enzyme involved in post-translational protein modification, plays a role in oxygen-dependent proline hydroxylation of HIF-α, IL-1 signaling, and neutrophil degranulation (47). A recent study has demonstrated that Uchl5 can deubiquitinate and activate NLRP3 in HCV-infected cells, leading to IL-1β maturation and secretion (48). However, its role in NLRP3 caused by cardiac I/R has not been reported. Interestingly, we found for the first time that Uchl5 was significantly elevated after I/R and could be inhibited by HS, exhibiting a comparable expression pattern to that of the NLRP3 inflammasome, which may partly account for the inhibitory effect of HS on NLRP3 inflammasome. The function of Lmo7, the LIM domain protein, is to bind actin or metal ion to regulate adherens junction and actin cytoskeleton, which has been identified to be induced by TGF-β and served as a negative feedback regulator of TGF-β signal (49). As the TGF-β signal implicates the release of inflammatory factors and the expression of adhesion molecules at the initial stage of I/R, it is speculated that Lmo7 may be associated with I/R-induced inflammation.

Oxidative stress and activation of NLRP3 inflammasome are both accompanied by mitochondrial damage, and are together implicated in the pathogenesis of myocardial I/R injury (50). The results of TEM validate this finding, demonstrating that I/R led to mitochondrial swelling and vacuolization, while HS notably attenuated the destruction of myocardial ultrastructure. Additionally, HS also improved mitochondrial ETC activities by elevating the expression of the subunits of complexes, such as COX7A and ATP5A.

The protective effect of HS on mitochondria may be related to the upregulation of Bdh1, Ckmt2, and COX7A. It is noteworthy that Bdh1, Ckmt2, and COX7A are mainly located in mitochondria and are closely related to mitochondrial function. These proteins were downregulated by I/R and remarkably reversed by HS. Bdh1, an enzyme located in the mitochondrial matrix, was mainly involved in ketone body metabolism, fatty acid beta-oxidation, and acyl-CoA metabolic process. Recent studies have reported that cardiac Bdh1 over-expression ameliorated pressure overload-induced oxidative stress and cardiac remodeling (51). Ckmt2 is the creatine kinase mitochondrial 2 that participates in the phosphocreatine pathway to generate ATP, calcium signaling, NOD-like receptor signaling pathway, etc. Ckmt2 over-expression was shown to restore mitochondrial function and protect against I/R and H/R injury (52, 53). It has been documented that blocking key enzymes associated with mitochondrial ETC significantly induces a surge in ROS production, leading to the spontaneous activation of NLRP3 inflammasome (5). In line with the report, as an important subunit of mitochondrial ETC, the low expression of COX7A leads to the leakage of electrons from ubiquinol to cytochrome c and from cytochrome c to oxygen, thus becoming the main source of ROS (54, 55). Excitingly, the reduction of COX7A, as well as Complex IV activity, induced by I/R was remarkably restored by HS. Furthermore, the activities of Complexes I, II, and V were also recovered by HS after I/R, indicating the pivotal role of mitochondrial ETC in the inhibitory effect of HS on ROS generation and NLRP3 inflammasome activation. These data highlight the involvement of mitochondrial function in the beneficial role of HS.

**FIGURE 10:**
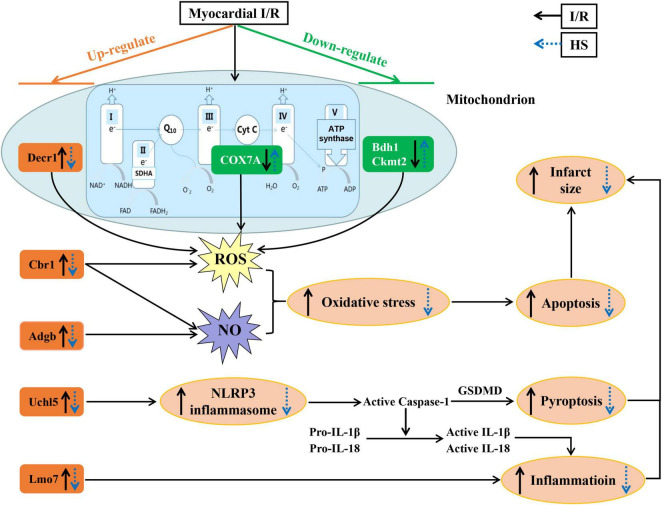
A diagrammatic sketch displaying the pathways that lead to the protective effect of Herba Siegesbeckiae (HS) on Ischemia/Reperfusion (I/R)-induced cardiac injury. I/R upregulates the expression of Adgb, Cbr1, Decr1, Uchl5, and Lmo7, and downregulates the expression of Bdh1, Ckmt2, and COX7A, resulting in oxidative stress and NLRP3 inflammasome activation with enhancement of inflammation, pyroptosis, apoptosis, and infarct size. Nevertheless, HS pre-treatment reverses all the above alterations. The cardioprotective role of HS relies on the inhibition of oxidative stress and NLRP3 inflammasome, and the restoration of mitochondrial functions.

As concluded in Figure 10, this is the first study to demonstrate the potential role of HS aqueous extract in protecting against cardiac I/R injury, as evidenced by the diminishment of infarct size and the preservation of myocardial morphology. The cardioprotective effect of HS may rely on the mitigation of oxidative stress, inhibition of NLRP3 inflammasome, and restoration of mitochondrial function, via the regulation of Adgb, Cbr1, Decr1, Eif5, Uchl5, Lmo7, Bdh1, Ckmt2, COX7A, and RT1-CE1 expression. These findings provide novel molecular targets and therapeutic options against cardiac I/R injury. Nevertheless, it is difficult to administer HS prior to I/R in a clinical setting, which limits the application of HS to a large extent. For more consistent clinical application, further studies are essential to isolate the bioactive compounds of HS, which can be administrated intravenously during ischemia or reperfusion. In addition, we will further explore the cardioprotective targets of the bioactive compounds of HS and clarify the potential signaling mechanism.

## Data Availability Statement

The datasets presented in this study can be found in online repositories. The names of the repository/repositories and accession number(s) can be found below: ProteomeXchange, PXD032886.

## Ethics Statement

The animal study was reviewed and approved by the Animal Care and Use Committee of Dongzhimen Hospital Affiliated to Beijing University of Chinese Medicine.

## Author Contributions

SL and H-CS designed the study and provided instructions for all experiments. XHW, YZW, HEP, QZ, KH, GYX, and HX performed the experiments and analyzed the data. All authors listed have read and approved this manuscript.

## Conflict of Interest

The authors declare that the research was conducted in the absence of any commercial or financial relationships that could be construed as a potential conflict of interest.

## Publisher’s Note

All claims expressed in this article are solely those of the authors and do not necessarily represent those of their affiliated organizations, or those of the publisher, the editors and the reviewers. Any product that may be evaluated in this article, or claim that may be made by its manufacturer, is not guaranteed or endorsed by the publisher.
