# Supplementary figures and images for: Proteomics Revealed That Mitochondrial Function Contributed to the Protective Effect of Herba Siegesbeckiae Against Cardiac Ischemia/Reperfusion Injury
Source: Front Cardiovasc Med. 2022 Jul 6;9:895797. doi: 10.3389/fcvm.2022.895797 (PMC9299383; doi:10.3389/fcvm.2022.895797)

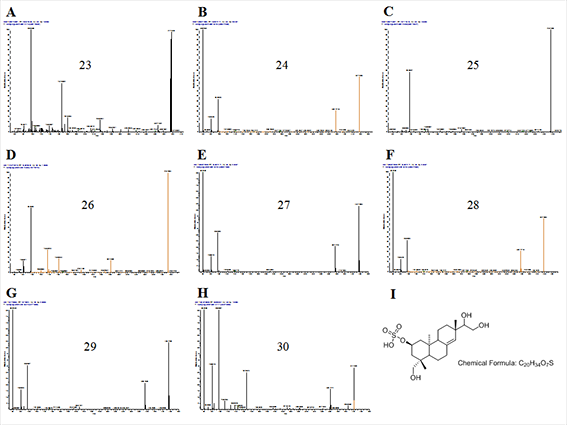

Supplement: Supplementary file 1 [file Image_1.TIF]

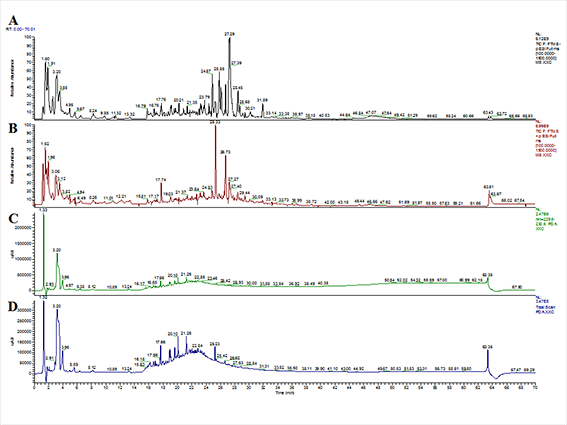

Supplement: Supplementary file 2 [file Image_2.TIF]

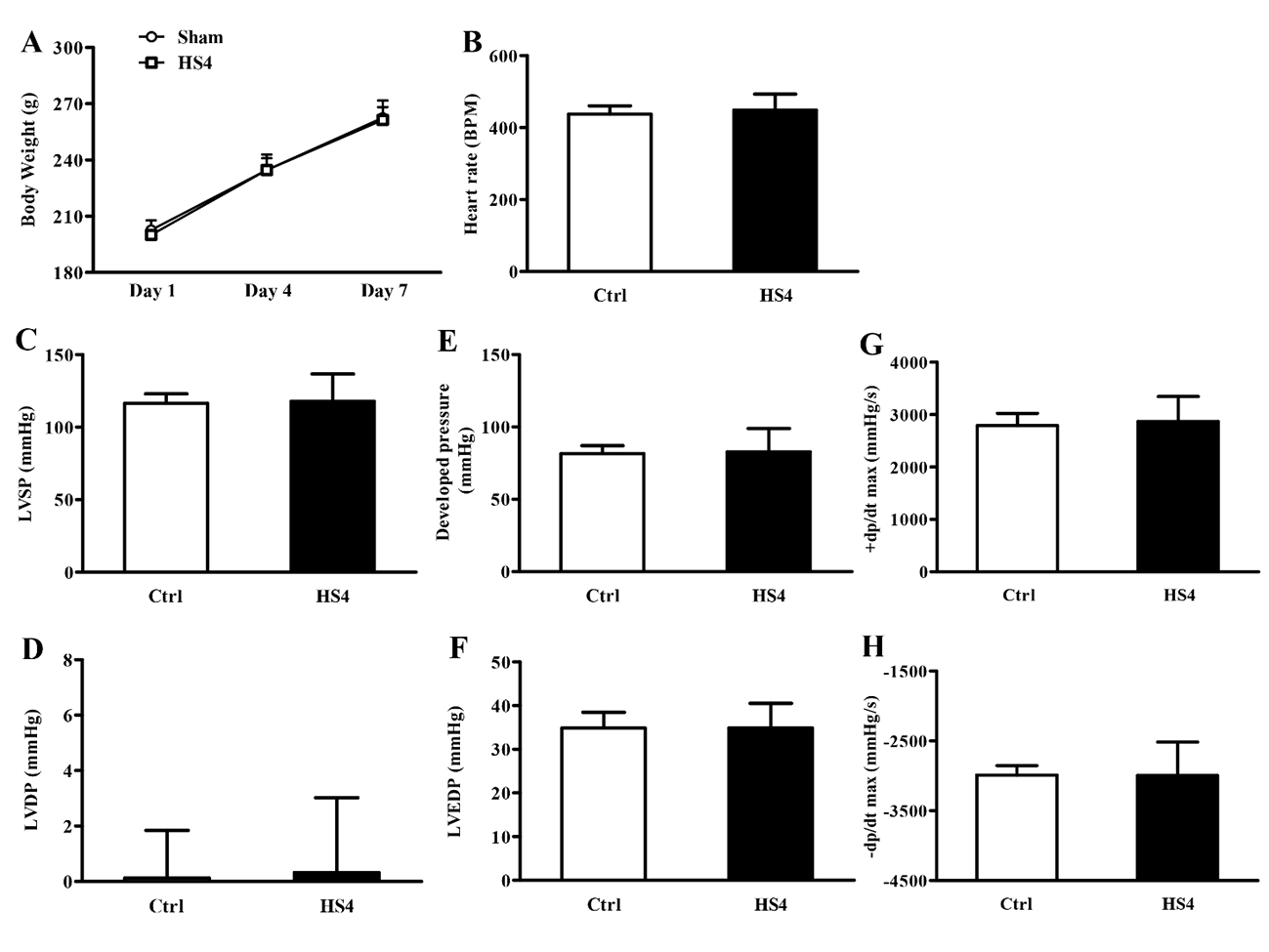

Supplement: Supplementary file 3 [file Image_3.TIF]

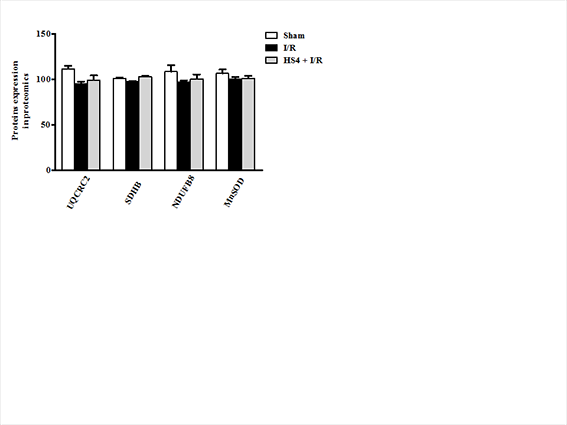

Supplement: Supplementary file 4 [file Image_4.TIF]
